# Common Carp *mef2* Genes: Evolution and Expression

**DOI:** 10.3390/genes10080588

**Published:** 2019-08-01

**Authors:** Mei He, Di Zhou, Nai-Zheng Ding, Chun-Bo Teng, Xue-Chun Yan, Yang Liang

**Affiliations:** 1 National and Local Joint Engineering Laboratory for Freshwater Fish Breeding, Heilongjiang River Fisheries Research Institute, Chinese Academy of Fishery Sciences, Harbin 150070, China; 2 College of Life Science, Northeast Forestry University, Harbin 150040, China; 3 College of Life Science, Shandong Normal University, Jinan 250014, China

**Keywords:** MEF2, *Cyprinus carpio*, evolution, expression, ohnologs

## Abstract

The MEF2 (myocyte enhancer factor 2) family belongs to the MADS-box superfamily of eukaryotic transcription factors. The vertebrate genes compose four distinct subfamilies designated MEF2A, -B, -C, and -D. There are multiple *mef2* genes in the common carp (*Cyprinus carpio*). So far, the embryonic expression patterns of these genes and the evolution of fish *mef2* genes have been barely investigated. In this study, we completed the coding information of *C. carpio mef2ca2* and *mef2d1* genes via gene cloning and presented two mosaic *mef2* sequences as evidence for recombination. We also analyzed the phylogenetic relationship and conserved synteny of *mef2* genes and proposed a new evolutionary scenario. In our version, MEF2B and the other three vertebrate subfamilies were generated in parallel from the single last ancestor via two rounds of whole genome duplication events that occurred at the dawn of vertebrates. Moreover, we examined the expression patterns of *C. carpio mef2* genes during embryogenesis, by using whole-mount in situ hybridization, and found the notochord to be a new expression site for these genes except for *mef2ca1&2*. Our results thus provide new insights into the evolution and expression of *mef2* genes.

## 1. Introduction

**Figure 1:**
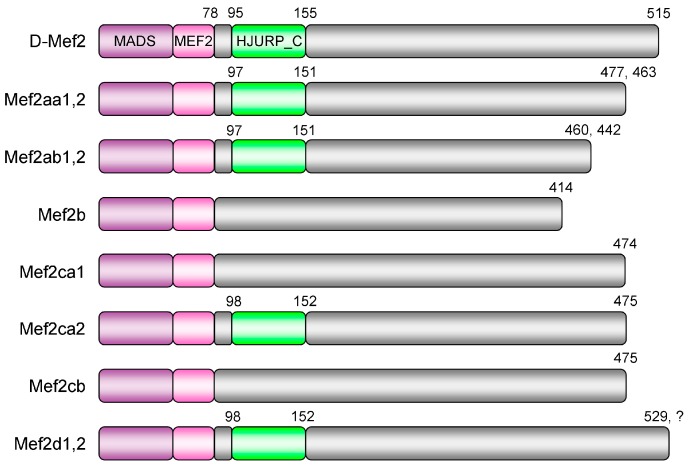
Domain structure of *Cyprinus carpio* Mef2 (myocyte enhancer factor 2) proteins. The N-terminal region of each Mef2 protein harbors the evolutionarily conserved MADS-box and MEF2 domains. The third homologous structure located in the highly variable C-terminal region, namely the HJURP_C domain, is not identified in Mef2b, Mef2ca1, and Mef2cb by Conserved Domain Search. The numbers indicated are amino acid positions of one isoform. D-Mef2 represents the Mef2 protein of *Drosophila melanogaster*. The ohnologs with the same structural feature are labeled together as "1,2". The question mark denotes that the protein sequence of Mef2d2 is incomplete at present.

The MEF2 (myocyte enhancer factor 2) family belongs to the MADS-box superfamily of eukaryotic TFs (transcription factors) that is named after the first four members to be recognized: MCM1 (minichromosome maintenance 1), AGAMOUS, DEFICIENS, and SRF (serum response factor) [1,2]. The family is now classified as MEF2-like/Type II, in parallel with SRF-like/Type I. Broadly speaking, included are products of the single invertebrate *MEF2* genes and the four vertebrate paralogs designated *MEF2A*, *-B*, *-C*, and *-D* [2,3]. The extreme N-terminus of each MEF2 protein harbors the evolutionarily conserved MADS-box motif. Directly adjacent is the MEF2 domain (Figure 1), another homologous stretch unique to this family. Both structures are required for efficient DNA binding, member dimerization and partner interaction [2,4]. In contrast, the C-terminal segment serves as the transactivation domain and allows high genetic variation and complex alternative splicing [3,5].

The vertebrate MEF2s are expressed in distinct yet overlapping patterns during embryonic and adult life. As central regulators, these TFs are implicated in the genesis and maintenance of diverse tissues, particularly muscle, nerve and the immune system [4,6]. Such activities are achieved via binding as homo- or hetero-dimers to the consensus element YTA(A/T)_4_TAR situated in the regulatory regions of the target genes, in response to various signaling pathways, such as Ca^2+^ and MAPK (mitogen-activated protein kinase) [3,6,7]. In different cell types, MEF2s can partner with different regulators, as exemplified by the members of the MYOD family of bHLH (basic helix-loop-helix) TFs that control skeletal myogenesis [7,8].

Notably, the vast majority of teleosts possess an exceptional number of *mef2* genes (Table 1). For example, in zebrafish (*Danio rerio*) that belongs to the family Cyprinidae, there are two versions for *mef2a* and *mef2c*, resulting in six genes, namely, *mef2aa*, *-ab*, *-b*, *-ca*, *-cb*, and *-d* [9]. The embryonic expression patterns of these genes have been assessed [10,11,12,13,14,15,16,17,18,19]. However, little is known about *mef2ab* and *mef2b* as yet, except that neither was detectable in somites at 48 hpf (hours post-fertilization) [13]. In addition, unlike the tetrapod counterparts [8,20], the evolution of fish *mef2* genes has been barely studied, thus far.

The common carp (*Cyprinus carpio*), another Cyprinidae member, is a freshwater species that is native to Asia and Europe, but has been introduced worldwide [21,22]. Farmed for food, ornamental or recreation, *C. carpio* is of economic significance in China, and accounts for ~10% of annual freshwater aquaculture production in the world [23,24,25]. Interestingly, it is also emerging as an alternative fish model, which has been intensively used, for instance, in immunology research [26,27,28,29,30,31]. To date, however, *C. carpio mef2* genes remain largely unexplored. 

Here, we completed the coding information of *C. carpio mef2ca2* and *mef2d1* genes and identified two mosaic *mef2* sequences that are indicative of recombination. We also analyzed the phylogenetic relationship and conserved synteny of *mef2* genes and proposed a new evolutionary scenario. In our version, the four vertebrate subfamilies were generated in parallel from the single last ancestor via two rounds of WGD (whole genome duplication) events that occurred at the dawn of vertebrates. Moreover, we examined the embryonic expression patterns of *C. carpio mef2* genes and found the notochord to be a new expression site for these genes except for *mef2ca1&2*. 

## 2. Materials and Methods

### 2.1. Fish Husbandry, Embryo Manipulation and Tissue Collection

All animal experiments were conducted on wild common carp bred in Heilongjiang River Fisheries Research Institute, as formerly reported [32]. Embryos were grown at 23 °C until the intended somite stages or time post-fertilization. For WISH (whole-mount in situ hybridization), the dechorionated samples were fixed with 4% paraformaldehyde at 4°C overnight, dehydrated in methanol, and stored at −20°C prior to use. Tissues, including brain, heart, and skeletal muscle, were collected by dissection from three 1-year-old individuals, frozen instantly in liquid nitrogen, and stored at −80°C prior to use. This study was approved by the Animal Care and Use Committee of Heilongjiang River Fisheries Research Institute of the Chinese Academy of Fishery Sciences (HSY201813K1-1), and was carried out in accordance with the approved guidelines.

### 2.2. RNA Extraction, cDNA Synthesis and Gene Cloning

Total RNA was extracted from *C. carpio* tissues using the TriPure Isolation Reagent (Roche, Basel, Switzerland) and treated with DNase I (TaKaRa, Dalian, China) to eliminate possible genomic DNA contamination. RNA quality was checked via formaldehyde denaturing gel electrophoresis, while RNA purity and quantity were measured by a NanoDrop 2000 Spectrophotometer (Thermo Scientific, Waltham, USA). Subsequently, cDNAs (20 μL/tube) were synthesized by Superscript III Reverse Transcriptase (Invitrogen, Carlsbad, USA) via incubation at 50 °C for 60 min. To amplify the entire CDS (coding sequence) of each desired *mef2* gene, conventional PCR (50 μL/tube) was run with gene-specific primer pairs (Table S1) using *Pyrobest* DNA Polymerase (TaKaRa). The program was set on a Bio-Rad T100 Thermal Cycler as follows—94°C 2 min; 94°C 30 s, 58°C 30 s, 72°C 2 min, 27 cycles; 72°C 2 min. Target products were purified by using the Wizard® SV Gel and PCR Clean-Up System (Promega, Madison, USA), and then ligated to the pEASY-T3 vector (TransGen, Beijing, China) for sequencing (Invitrogen, Shanghai, China). The obtained sequences of *mef2ca* and *mef2d1* were deposited in GenBank as MK946444 and MK946445, respectively.

### 2.3. Phylogenetic Reconstruction, Recombination Detection and Synteny Analysis

**Figure 2:**
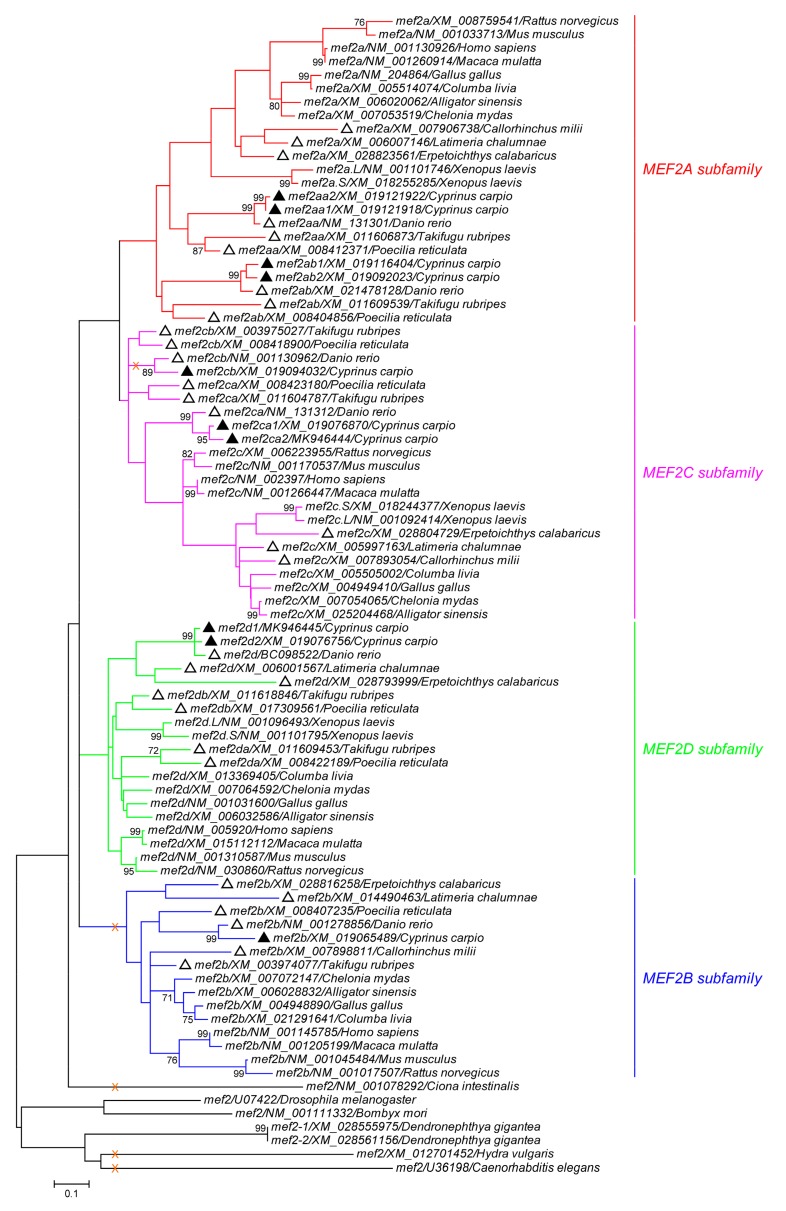
Phylogeny of the MEF2 family. The maximum likelihood tree was constructed based on the *mef2* coding region 1–258. Information of each sequence used is given as name/accession/species. The scale bar indicates the number of nucleotide substitutions per site. Branches supported by >70% bootstrap value (1000 replicates) are labeled, and those lacking the HJURP_C domain are indicated with "X". The four vertebrate MEF2 subfamilies are highlighted. The sequences of *C. carpio* and the other fish species are marked with "▲" and "△", respectively.

Full-length *mef2* CDSs of representative species (Figure 2), alongside with those of *C. carpio*, were retrieved from GenBank. A dataset of 83 sequences (Figure 2) was then compiled and subjected to alignment by CLUSTAL W [33]. However, considering that the C-terminal region is too divergent to be informative, only the first 258 bp segment was reserved for phylogenetic reconstruction. As done previously [34,35], the ML (maximum likelihood) tree was drawn by MEGA6 [36] with 1000 bootstrap replicates, under the best-fit nucleotide substitution model GTR + G + I. Moreover, recombination detection was applied using methods described previously [37,38], while conserved synteny analysis was conducted based on the Synteny Database [39] and the *C. carpio* genome [23].

### 2.4. Whole-Mount In Situ Hybridization

The expression patterns of *C. carpio mef2* genes during embryogenesis were examined by WISH following the standard zebrafish protocol [40,41]. The gene-specific riboprobes were prepared as before [42]. Briefly, each gene segment (~200 bp) was amplified from tissue cDNAs with the corresponding primers listed in Table S1, and then cloned into pEASY-T3 for sequencing. Afterwards, each was retrieved from the verified plasmid with T7 and SP6 primers for template linearization, and then made into a digoxigenin-tagged probe using the DIG RNA Labeling Kit (Roche). WISH images were captured on an Olympus SZX7 Zoom Stereo Microscope.

## 3. Results

### 3.1. Multiple mef2 Genes in C. carpio

Strikingly, there are 12 *mef2* genes predicted in the haploid genome of *C. carpio* (Table 2). Compared with *D. rerio* genes (Table 1), every ortholog is present in duplicate, albeit both *mef2b* and *mef2cb* have one version that appears to be a pseudogene. Herein, the two versions of the other four paralogs were provisionally distinguished by Arabic numerals. Moreover, although the genomic information of *C. carpio mef2* genes is not complete at present, it is highly possible that the new ohnologs are located on the same chromosome (Table 2).

Notably, for *C. carpio*, there are two validated entire CDSs tagged as *mef2a* and *mef2c* available in GenBank under the Accession numbers AB012884 and AB012885, respectively. According to sequence homology, the latter is *mef2ca1*, while the former appears to be a recombinant between *mef2aa1* and *mef2aa2*. The recombination event was suggested to occur at coding position 395 by the BootScan method (*p* = 8.44E−03). The mosaic *mef2aa*/AB012884 was identical to *mef2aa1*/XM_019121918 before the breakpoint, but shared higher similarity with *mef2aa2*/XM_019121928 in the remaining region (Figure S1A).

In addition, sequence alignments revealed that the sequences of *mef2ca1*, *mef2ca2* and *mef2d2* predicted from the genome were all incomplete, with the coding regions 259-399, 1-54 and 871-1199 being missing, respectively. Meanwhile, as suggested by BLAST, sequence fragments of *mef2d1*, except the coding region 987-1199, could be mapped to three different genomic assemblies (Table 2).

Because AB012885 has the complete coding information of *mef2ca1*, we attempted to clone the entire CDSs for the other three genes. Surprisingly, the predominant forms obtained turned out to be *mef2d1* (MK946445) and a chimeric *mef2ca* (MK946444). This time, the probable breakpoint was located at coding position 673 (*p* = 3.81E−07). The chimera *mef2ca*/MK946444 more resembled *mef2ca2*/XM_019076871 before this site, but was closer to *mef2ca1*/AB012885 thereafter (Figure S1B). Nevertheless, for the missing part (coding region 1-54) of *mef2ca2*, MK946444 can now be referred to.

Then, the deduced amino acid sequences were subjected to Conserved Domain Search. As illustrated in Figure 1, all *C. carpio* members have the characteristic MADS-box and MEF2 domains. Besides, the MEF2 family has also been provided with a third homologous structure known as the HJURP_C domain, standing for the Holliday junction recognition protein C-terminal repeat. The lack of HJURP_C is a common feature of the vertebrate MEF2B subfamily, as represented by *C. carpio* Mef2b here. Surprisingly, the domain was not recognized in the corresponding regions of both Mef2cb and Mef2ca1, due to several indels and two substitutions (E113G and R117C), respectively. 

### 3.2. Phylogeny and Conserved Synteny of mef2 Genes

To explore the evolutionary relationship of *mef2* genes, an ML phylogeny (Figure 2) was constructed based on the CDS segment 1-258, which is just upstream of the first alternative splicing location shared by vertebrate subfamilies except for MEF2B. Consistent with previous findings [8], within the vertebrate lineage, MEF2A and MEF2C are sister clades, whereas MEF2B is the most distant one, though topologies were not well supported (bootstrap value < 70%). However, judging from the branch depth, MEF2A experienced more genetic diversity in this region than the other three subfamilies, with *mef2a*/XM_008759541/*Rattus norvegicus* being the most divergent one.

In addition to *C. carpio*, a cartilaginous fish and five bony fish were included in the phylogenetic analysis. Figure 2 shows that only *C. carpio* and *D. rerio* were invariably tied to each other, while *Callorhinchus milii*, the cartilaginous species carrying three *mef2* genes, always clustered with the tetrapods. Interestingly, the other four bony species exhibited incongruent topologies. Those falling into the tetrapod group comprised *mef2a* and *mef2c* of *Latimeria chalumnae* and *Erpetoichthys calabaricus*, *mef2da* and *mef2db* of *Poecilia reticulata* and *Takifugu rubripes*, as well as *mef2b* of *T. rubripes*.

**Figure 3:**
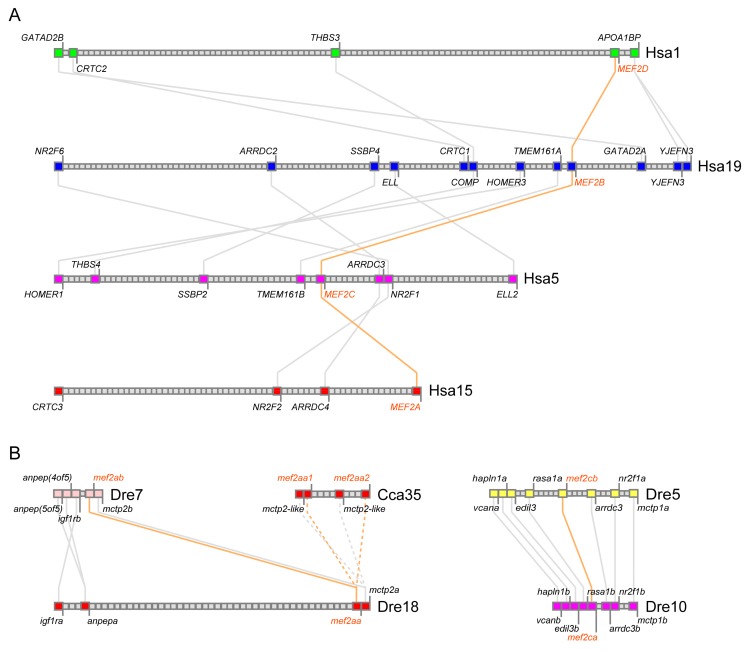
Conserved synteny analysis with *mef2* genes of *Homo sapiens* (**A**), *Danio rerio* and *Cyprinus carpio* (**B**). In each species, squares of the same color represent genes belonging to a syntenic cluster, with gene names indicated beside. Species and chromosome number are labeled on the right of each cluster (Hsa: *H. sapiens*; Dre: *D. rerio*; Cca: *C. carpio*). Solid and dashed lines connect paralogous and orthologous gene pairs, respectively. For simplicity, lines between Hsa1 and Hsa5, Hsa1 and Hsa15, as well as Hsa19 and Hsa15 are not shown. All *mef2* genes and their connections are highlighted in orange.

For more information to infer the evolutionary history of *mef2* genes, conserved synteny was assessed with the genomes of human, *D. rerio* and *C. carpio*. Figure 3A shows the four syntenic segments on different human chromosomes —Hsa1, Hsa5, Hsa15, and Hsa19, with each harboring a *MEF2* gene. This indicates that the four human paralogs come from the two-round vertebrate WGD (1R and 2R) [43,44]. Based on common genes in the neighborhood, *MEF2D* is much closer to *MEF2B* than to the others, while *MEF2B* and *MEF2C* share the highest synteny. These suggest that the 2R ohnologous pairs are *MEF2A* and *MEF2C* vs. *MEF2B* and *MEF2D*, with *MEF2B* and *MEF2C* being closer to the original 1R copies, respectively. 

In *D. rerio*, *mef2aa* and *mef2ab* also lie on the syntenic segments (Dre18 and Dre7), so do *mef2ca* and *mef2cb* (Dre10 and Dre5) (Figure 3B), supporting that *D. rerio mef2* genes experienced the teleost-specific 3R WGD (Ts3R) [44,45]. For *C. carpio*, the pair of *mef2aa1* and *mef2aa2* was analyzed as a representative. According to the present genomic assembly, the two genes are close to each other on Cca35 and exhibit conserved synteny as well (Figure 3B), suggesting that *C. carpio mef2* genes followed the carp-specific 4R WGD (Ca4R) occurring after the separation of *D. rerio* [23]. 

### 3.3. Embryonic Expression Patterns of C. carpio mef2 Genes

To examine the spatial-temporal expression patterns of *C. carpio mef2* genes during early embryogenesis, WISH was carried out with the gene-specific antisense RNA probes. However, it should be noted that for the new ohnologs, although the probe was designed to target one version, the other one would be detected as well due to the strong sequence similarity (~95%) between them. Here, the two versions together were denoted as “*1&2*”. Moreover, because we did not obtain any amplicon of *mef2ab1* or *mef2ab2* in several trials, these two genes were not included in this section.

**Figure 4:**
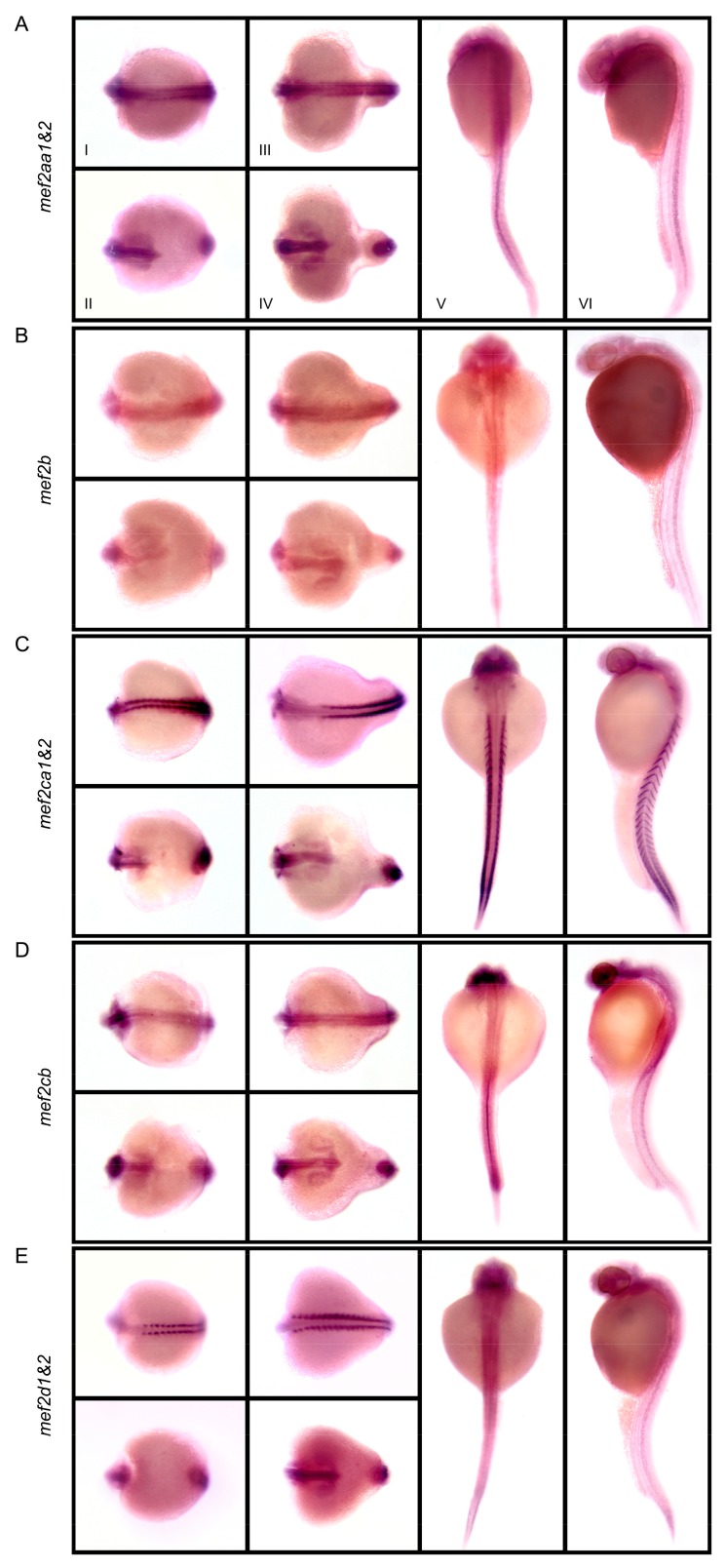
Embryonic expression patterns of *Cyprinus carpio mef2* genes, including *mef2aa1&2* (**A**), *mef2b* (**B**), *mef2ca1&2* (**C**), *mef2cb* (**D**), and *mef2d1&2* (**E**). Whole-mount in situ hybridization was conducted on *C. carpio* embryos at 8-somite stage (I, II), 18-somite stage (III, IV) and 24 hpf (V, VI). The two ohnologous versions were detected together, thus being denoted as "*1&2*". I and III: Dorsal view, anterior to the left; II and IV: Ventral view, anterior to the left; V: Dorsal view, anterior up; VI: Lateral view, anterior up.

Figure 4 shows that, at different somite stages, expression patterns of the analyzed *mef2* genes overlapped in the developing brain and eyes, the two parallel cords of adaxial cells flanking the notochord, as well as the tail bud, but differed in the extending notochord. In sharp contrast to the other *mef2* genes, transcripts of *mef2ca1&2* and *mef2d1&2* were barely detectable in the notochord. However, *mef2d1&2* exhibited a biphasic expression pattern in the trunk. At 24 hpf, it switched to the expression pattern similar to *mef2aa1&2*, *mef2b* and *mef2cb*, which retained only clear notochord signal. Notably, *mef2ca1&2* was the only *mef2* gene product present at high levels in the bilateral heart fields, visible as two dots (Figure 4C, dorsal view), and at myotome boundaries as repetitive chevrons (Figure 4C, lateral view). In addition, *mef2cb* was also detected in the pronephric duct (Figure 4D, lateral view).

## 4. Discussion

Based on phylogenetic relationships and the difference in HJURP_C, Wu et al. [8] proposed that the four vertebrate *MEF2* paralogs were generated from a common ancestor via three rounds of duplication. Given that MEF2B is the most distant one and original MEF2 proteins do not have HJURP_C, they presumed that the origin of MEF2B is more ancient than those of the other three that contain HJURP_C. According to the presence of two distinct MEF2-type genes in an invertebrate species *Nematostella vectensis*—one has HJURP_C, but the other does not, they further presumed that the first round of duplication occurred before the origin of vertebrates, producing two copies of *MEF2* genes—one finally became extant *MEF2B*, whereas the other underwent the insertion of HJURP_C followed by two rounds of duplication to produce *MEF2D*, *-A* and *-C* near the origin of vertebrates.

However, it should be pointed out that the available sequence of the hypothetical MEF2 protein (XP_001627736) of *N. vectensis* thought to lack HJURP_C is partial, which does not cover the whole C-terminal region next to the MEF2 domain. Thus, it cannot be asserted that HJURP_C is absent in this protein. In fact, HJURP_C is present in both of the two MEF2s (XP_028411776 and XP_028416957) of *Dendronephthya gigantea*, another Anthozoa species. Besides, unlike their depiction [8], HJURP_C is also possessed by the single MEF2 (AAA20463) of *Drosophila melanogaster* (Figure 1). Nevertheless, HJURP_C is missing in MEF2s of *Hydra vulgaris* (XP_012556906), *Caenorhabditis elegans* (AAA79336) and *Ciona intestinalis* (NP_001071760).

Given that independent loss is more feasible than independent insertion and *C. carpio* Mef2cb (Figure 1) might offer an example, the likely evolutionary episode of HJURP_C in the MEF2 family is that the insertion event took place around the onset of Cnidaria, and in the following course, it was not retained by some invertebrate species like the three mentioned above, as well as the entire vertebrate MEF2B subfamily (Figure 2). Notably, HJURP_C is also not identified in the Mef2cb proteins of *D. rerio* (NP_001124434) and *Carassius auratus* (XP_026107184); however, this is not the case with other teleosts, such as *P. reticulata* (XP_008421402) and *T. rubripes* (XP_011603089), suggesting that this loss event might be specific to the Cyprinidae family. 

With respect to the evolution of the MEF2 family, we favor another scenario: To produce the four vertebrate paralogs, the single last ancestor containing HJURP_C just followed 1R and 2R that took place at the root of the vertebrate lineage [43,44]. Indeed, it is supported by the conserved synteny analysis conducted on human *MEF2* genes (Figure 3A). Notably, the four paralogs in *L. chalumnae* and *E. calabaricus*, two primitive fish species, could be classified into two groups—*mef2a* and *mef2c* vs. *mef2b* and *mef2d*, according to their behavior in the ML tree (Figure 2). Therefore, although MEF2B and MEF2D did not bifurcate from a single clade (Figure 2), the two are also sister lineages, just like MEF2A and MEF2C. Such relationships can also be inferred from the conserved synteny analysis (Figure 3A). Moreover, as MEF2B lacks the first alternative splicing event common to the other three [2], which involves part of HJURP_C, it is possible that MEF2B suffered a unique exon loss event around the origin and has been getting more divergent from the others ever since. 

Subsequently, Ts3R was fulfilled before the radiation of bony fish [44,45]. Later on, several teleost lineages, including carps (Ca4R), salmonids and some cichlids (Table 1), independently went through an additional round of WGD. Accordingly, the multiple *mef2* genes in *C. carpio* were derived from four rounds of WGD, namely, 1R, 2R, Ts3R, and Ca4R. Of note, some other lineages leading to species, such as *Saccharomyces cerevisiae*, *D. gigantea* and *Xenopus laevis* (Table 1) also experienced separate WGD events. 

After WGD, one fate confronted by duplicated genes is the loss of one copy via nonfunctionalization (or pseudogenization) due to the accumulation of deleterious mutations [44,46]. Indeed, both *mef2b* and *mef2cb* of *C. carpio* had one version pseudogenized after Ca4R (Table 2). Besides, after 2R, *mef2b* was lost in *X. laevis*, so was *mef2d* in *C. milii* and *Ornithorhynchus anatinus* (Table 1). Notably, after Ts3R, loss of different genes resulted in highly variable gene combinations (Table 1). It is intriguing that *mef2b* might be the only one to be always kept as a singleton in this round. Perhaps, *mef2b* had a high mutation rate at that time, which raised its proneness to pseudogenization.

Nevertheless, in another fate, both copies will survive, mainly via subfunctionalization by partitioning ancestral functions and/or neofunctionalization by acquiring novel functions [44,46,47]. Actually, in most vertebrate species, all of the four *MEF2* paralogs were preserved after 2R. This might be owing to their developmental roles that are conducive to vertebrate evolutionary innovations [48]. Indeed, MEF2C has been identified to be a central regulator of the multipotent neural crest cells, a novel cell type in vertebrates [3]. So far, the functions of individual MEF2 members have not been fully explored; however, functional diversification has been suggested by their distinct though overlapping expression patterns [6] (Figure 4).

Compared with the expression patterns of *D. rerio mef2* genes [12,15,16,17], a notable difference is the appearance of the notochord signals in all analyzed *C. carpio* genes except for *mef2ca1&2* (Figure 4). For the ohnologs generated from Ca4R, it should be noted that, because the two versions could not be distinguished by the riboprobe, it is unknown for now whether only one or both of them exploited the new expression site. Interestingly, a concomitant novel function for *C. carpio mef2* genes in the notochord might still be related to skeletal muscle regulation, because the teleost notochord can secrete Hedgehog signals to control the formation of the horizontal myoseptum and specify the fate of slow-twitch muscle [49]. 

For *mef2cb*, additional differences revealed by WISH comprise the absence in the heart fields and the presence in the pronephric duct (Figure 4D), suggesting a substantial function adjustment. However, similar to the ortholog in *D. rerio* [12], *mef2cb* was weakly stained in the myotomes at 24 hpf, despite the high signals there during somite stages (Figure 4D). Such a decrease also happened to *mef2aa1&2* (Figure 4A) and *mef2d1&2* (Figure 4E). Notably, *mef2b* is transcribed at basal levels during embryogenesis (Figure 4B). It might be common to different species [20] that *mef2b* has the lowest expression level among the members, though the expression patterns of *mef2ab* remain to be elucidated.

Furthermore, it is remarkable that carp can rapidly adapt to a new habitat after introduction, thus distributing worldwide now. A major contributor might be the four rounds of WGD events, because gene duplication provides an additional source of genetic material, which will lessen, or even free one copy from, the selective pressure confronted by the original copy, thereby increasing the adaptive genetic diversity [47]. Interestingly, the Ca4R event also prepares the material for recombination, as manifested by the two mosaic *mef2* sequences. Of note, the two sequences were independently isolated and there was no assembly issue in gene cloning, since *mef2aa*/AB012884 was retrieved by Kobiyama et al. [50] from a cDNA library clone covering the entire CDS, while *mef2ca*/MK946444 was amplified here with just one pair of primers (Table S1) targeting the whole CDS. Therefore, the two should be *bona fide* recombinants between the duplicates. 

The Ca4R ohnologs involved in recombination are close together on the same genomic assembly (Table 2); however, given the uncertainty, it cannot be excluded that they are on different chromosomes. Moreover, due to the lack of allelic information, it cannot be told here whether each event is reciprocal unequal crossover or unidirectional gene conversion. Nevertheless, the identification of two cases in one gene family suggests that recombination might be active in boosting the evolution of *C. carpio*, which renders a shortcut via exchanging genetic material between the ohnologs carrying distinct variations that have already been selected.

## Figures and Tables

**Table 1:** Example species with an exceptional number of *mef2* genes.

Species		Gene No.	Gene Name
Invertebrate			
	*Saccharomyces cerevisiae*	2	*RLM1, SMP1*
	*Dendronephthya gigantea*	2	*MEF2-1, MEF2-2* ^1^
Vertebrate			
cartilaginous fish	*Callorhinchus milii*	3	*mef2a, -b, -c*
bony fish	*Ictalurus* *punctatus*	5	*mef2aa, -ab, -b, -c, -d*
	*Danio rerio*	6	*mef2aa, -ab, -b, -ca, -cb, -d*
	*Poecilia reticulata*	7	*mef2aa, -ab, -b, -ca, -cb, -da, -db*
	*Haplochromis burtoni*	11	*mef2aa1, -aa2, -ab1, -ab2, -b1,-b2, -ca, -cb, -da1, -da2, -db* ^1^
	*Salmo salar*	12	*mef2a1, -a2, -b1, -b2, -ca1, -ca2,-cb1, -cb2, -da1, -da2, -db1, -db2* ^1^
amphibian	*Xenopus laevis*	6	*mef2a.L, -a.S, -c.L, -c.S, -d.L, -d.S*
mammal	*Ornithorhynchus anatinus*	3	*MEF2A, -B, -C*

^1^ Provisional name.

**Table 2:** Predicted *mef2* genes in *Cyprinus carpio.*

Gene ^1^	ID	Chr ^2^	Location
*mef2aa1*	109108809	35	NC_031731 (6415225..6510272)
*mef2aa2*	109108812	35	NC_031731 (6804879..6863032)
*mef2ab1*	109103080	29	NC_031725 (2966793..2992778)
*mef2ab2*	109076298	Un	LN594175 (30006..48944)
*mef2b pseudo*	109047862	44	NC_031740 (10912568..10929359)
*mef2b*	109047821	44	NC_031740 (11026408..11033466)
*mef2ca1*	109059697	Un	LN591062 (partial)
*mef2ca2*	109059699	Un	LN591062 (partial)
*mef2cb pseudo*	109078847	Un	LN594801 (406945..466454)
*mef2cb*	109078846	Un	LN594801 (519901..748916)
*mef2d1* ^3^	?	?	LN591056 + NC_031744 + NC_031735 (partial)
*mef2d2*	109059568	Un	LN591056 (partial)

^1^ Provisional name. ^2^ Chr, chromosome; Un, unplaced scaffold. ^3^ Fragmented into 109059569 (Un), 109049995 (Chr 48) and 109112856 (Chr 35).

## Supplementary Materials

The following are available online at https://www.mdpi.com/2073-4425/10/8/588/s1, Figure S1: Evidence for recombination between the *mef2* ohnologs of *C. carpio*, Table S1: Primers used.
