# Supplementary material for: Common Carp mef2 Genes: Evolution and Expression
Source: Genes (Basel). 2019 Aug 1;10(8):588. doi: 10.3390/genes10080588 (PMC6723361; doi:10.3390/genes10080588)
Supplement: Supplementary file 1 [file genes-10-00588-s001.zip › Supplementary files/Figure S1.doc]

**Figure S1.** Evidence for recombination between the *mef2* ohnologs of *C. carpio*. BootScan analyses with *mef2aa*/AB012884/*Cyprinus carpio* (**A**), *mef2ca*/MK946444/*Cyprinus carpio* (**B**), and their parent sequences. In each plot, the y-axis gives the percentage of permutated trees using a sliding window of 400 bp and a step size of 50 bp. Two *Danio rerio* sequences, *mef2aa/*NM_131301 and *mef2ca/*NM_131312 were used as the out-group, respectively.
