# Supplementary material for: Common Carp mef2 Genes: Evolution and Expression
Source: Genes (Basel). 2019 Aug 1;10(8):588. doi: 10.3390/genes10080588 (PMC6723361; doi:10.3390/genes10080588)
Supplement: Supplementary file 1 [file genes-10-00588-s001.zip › Supplementary files/Table S1.doc]

**Table S1.** Primers used

| **Gene** | **Sequence (5′-3′)** | **RefSeq** |
| --- | --- | --- |
| gene cloning |  |  |
| *mef2ca* | F: ATGGGGAGGAAAAAGATTC | LN591062 |
|  | R: CGTGATCACGAGGCCCAGCC |  |
| *mef2d* | F: TGCCTCGAGAAATTTTGACC | LN591056 |
|  | R: TCAGCGTTTATGTGACCCAG | LN591056, NC_031735 |
| Riboprobe preparation | |  |
| *mef2aa* | F: TTCTCCATGCATGTGGCTGT | AB012884 |
|  | R: GCCCGGCAGACACTAAGTT |  |
| *mef2b* | F: TCTCATCAGGAGGCCCACCAG | XM_019065489 |
|  | R: TCTTCCATTGGTGAGTGCTGC |  |
| *mef2ca* | F: GCTCCACCCTCTCACTGCC | AB012885 |
|  | R: AGTCGATGCGGTGCTCCTC |  |
| *mef2cb* | F: AGCGACGCCTACTCTACCG | XM_019094032 |
|  | R: GCTGCTGCCATCCACTCA |  |
| *mef2d* | F: GTGCAGATCTCACAGCCCTC | MK946445 |
|  | R: AGGGATCGTTCCCACGGGC |  |
